# Gasdermin B expression predicts poor clinical outcome in HER2-positive breast cancer

**DOI:** 10.18632/oncotarget.10787

**Published:** 2016-07-22

**Authors:** Marta Hergueta-Redondo, David Sarrio, Ángela Molina-Crespo, Rocío Vicario, Cristina Bernadó-Morales, Lidia Martínez, Alejandro Rojo-Sebastián, Jordi Serra-Musach, Alba Mota, Ángel Martínez-Ramírez, Maria Ángeles Castilla, Antonio González-Martin, Sonia Pernas, Amparo Cano, Javier Cortes, Paolo G. Nuciforo, Vicente Peg, José Palacios, Miguel Ángel Pujana, Joaquín Arribas, Gema Moreno-Bueno

**Affiliations:** ^1^ Biochemistry Department, Universidad Autónoma de Madrid (UAM), Instituto de Investigaciones Biomédicas “Alberto Sols” (CSIC-UAM), IdiPAZ, Madrid, Spain; ^2^ Preclinical Oncology Program, Vall d'Hebron Institute of Oncology (VHIO), Universitat Autònoma de Barcelona, Barcelona, Spain; ^3^ Pathology Department, MD Anderson Cancer Center, Madrid, Spain; ^4^ Breast Cancer and Systems Biology Unit, ProCURE, Catalan Institute of Oncology, IDIBELL, L'Hospitalet del Llobregat, Barcelona, Spain; ^5^ Translational Research Laboratory, MD Anderson Internacional Foundation, Madrid, Spain; ^6^ Cytogenetics Department, MD Anderson Cancer Center, Madrid, Spain; ^7^ Pathology Department, Hospital Universitario Virgen del Rocío, Sevilla, Spain; ^8^ Oncology Department, MD Anderson Cancer Center, Madrid, Spain; ^9^ Clinical Oncology Program, Vall d'Hebron Institute of Oncology (VHIO), Universitat Autonoma de Barcelona, Barcelona, Spain; ^10^ Oncology Department, Hospital Universitario Ramón y Cajal, Madrid, Spain; ^11^ Molecular Oncology Program, Vall d'Hebron Institute of Oncology (VHIO), Universitat Autònoma de Barcelona, Barcelona, Spain; ^12^ Pathology Department, Hospital Vall d'Hebron University, Barcelona, Spain; ^13^ Pathology Department, Hospital Universitario Ramón y Cajal, Madrid, Spain

**Keywords:** HER2-positive breast cancer, gasdermin B, clinical behaviour, predictive biomarker, resistance to therapy

## Abstract

Around, 30–40% of HER2-positive breast cancers do not show substantial clinical benefit from the targeted therapy and, thus, the mechanisms underlying resistance remain partially unknown. Interestingly, *ERBB2* is frequently co-amplified and co-expressed with neighbour genes that may play a relevant role in this cancer subtype. Here, using an *in silico* analysis of data from 2,096 breast tumours, we reveal a significant correlation between Gasdermin B (*GSDMB*) gene (located 175 kilo bases distal from *ERBB2*) expression and the pathological and clinical parameters of poor prognosis in HER2-positive breast cancer. Next, the analysis of three independent cohorts (totalizing 286 tumours) showed that approximately 65% of the HER2-positive cases have *GSDMB* gene amplification and protein over-expression. Moreover, GSDMB expression was also linked to poor therapeutic responses in terms of lower relapse free survival and pathologic complete response as well as positive lymph node status and the development of distant metastasis under neoadjuvant and adjuvant treatment settings, respectively. Importantly, GSDMB expression promotes survival to trastuzumab in different HER2-positive breast carcinoma cells, and is associated with trastuzumab resistance phenotype *in vivo* in Patient Derived Xenografts. In summary, our data identifies the *ERBB2* co-amplified and co-expressed gene *GSDMB* as a critical determinant of poor prognosis and therapeutic response in HER2-positive breast cancer.

## INTRODUCTION

The identification of molecular cancer drivers has considerably improved clinical outcomes in oncology. As such, inhibition of the human epidermal growth factor receptor 2 (HER2; *ERBB2* gene) represents a paradigm in breast cancer oncology. Over-expression of *ERBB2* occurs in approximately 20% of breast tumours [1, 2]. Thus, the amplification of chromosome 17q12-q21, which includes *ERBB2*, defines an intrinsic molecular cancer subtype associated with relatively aggressive tumour behaviour and poor clinical outcome [3]. However, the development of targeted anti-HER2 therapies based principally on humanized antibodies (such as trastuzumab, trastuzumab emtansine -T-DM1 or pertuzumab) and tyrosine kinase inhibitors (such as lapatinib) has dramatically improved the outcome of women with early and advanced stage HER2-positive breast cancer [4–11]. Several clinical trials have demonstrated that combination with chemotherapy in the neoadjuvant or adjuvant setting significantly improves clinical response without relevant toxicity [5, 13–16]. Despite the clinical benefit obtained from the above therapies, inhibition of HER2 alone is generally not sufficient. In fact, trastuzumab response rate is limited to approximately 15–26% [12, 13], which indicates the involvement of other molecules and/or signalling pathways that influence outcome in this setting [14].

Since the common 17q12-q21 amplicon includes several gene *loci*, *ERBB2*-co-amplified genes have been examined for their potential to influence cancer progression and/or therapeutic response [17, 18]. A minimal amplified region around *ERRB2* oncogene has been defined in several studies [19, 20], and some of the genes have been found to play a role in tumorigenesis [14, 21, 22]. These genes include *STARD3* and *GRB7*, whose products promote the proliferation of HER2-positive breast cancer cell lines [18]. Thus, GRB7 over-expression has been identified as an independent prognostic factor [23], and the co-amplification of *ERBB2* with the non-core gene topoisomerase II (*TOP2A*) predicts sensitivity to anthracycline therapy [21, 24, 25]. Together, these observations illustrate the importance of additional genes in the biology and clinical evolution of HER2-positive breast cancer.

Previous data demonstrated that *GSDMB* lies within the evolutionary recombination hotspot closely linked to the *ERBB2* amplicon [20, 26]. The gene product belongs to the family of gasdermins, which includes three other human members [27–31]. Although these other members have been implicated in the development and progression of some diseases [32, 33], the role of GSDMB in cancer is only now beginning to emerge [30, 31, 34]. We recently demonstrated that GSDMB over-expression promotes cell motility, invasion and metastasis of breast cancer cell lines, and, intriguingly, it was found over-expressed in breast tumour samples [34]; however, the potential link with breast cancer subtypes remained unexplored.

Here, through integrated gene expression and molecular analyses, we show robust associations between *GSDMB* amplification/expression and HER2-positive status. Most importantly, further analyses show that *GSDMB* gene and protein expression predicts poor clinical outcome in HER2-positive breast cancer treated, both in the neoadjuvant and adjuvant settings. Importantly, we corroborated that GSDMB expression is associated with trastuzumab resistance phenotype in HER2-positive breast carcinoma cells and in Patient Derived Xenografts. GSDMB increases cell growth and reduces apoptosis after trastuzumab treatment in breast cancer cells. Together, our data reveals GSDMB as a key prognostic and predictive biomarker in HER2-positive breast cancer, highlighting new opportunities for valuable combined therapies.

## RESULTS

### GSDMB over-expression is associated with poor prognosis of HER2-positive breast cancer

We have previously reported that relative high expression of *GSDMB*, but no other *GSDM* genes, in breast tumours is associated with poor survival in unselected breast cancer cases [34]. To assess further the molecular and clinical significance of this observation, two independent microarray expression datasets including a total of 2,096 cases were analysed [35, 36]. Overall, high *GSDMB* expression was correlated with HER2-positive status; according to the PAM50 classifier [37] or with the reported immunohistochemical results (Supplementary Table 1).

Next, the association between *GSDMB* over-expression and 17q12-q21 amplification was evaluated. Using copy number estimates from The Cancer Genome Atlas (TCGA) dataset [36], the *GSDMB* locus was shown to be amplified in 58 out of 526 (11%) tumours. Importantly, this alteration was observed only in tumours that also presented *ERBB2* amplification (58/67; 86%); thus, none of the 459 HER2-negative cases was identified as harbouring *GSDMB* amplification. Consistent with this observation, high *GSDMB* expression levels were associated with both *ERBB2* and *GSDMB* gene amplification (Supplementary Table 1).

**Figure 1:**
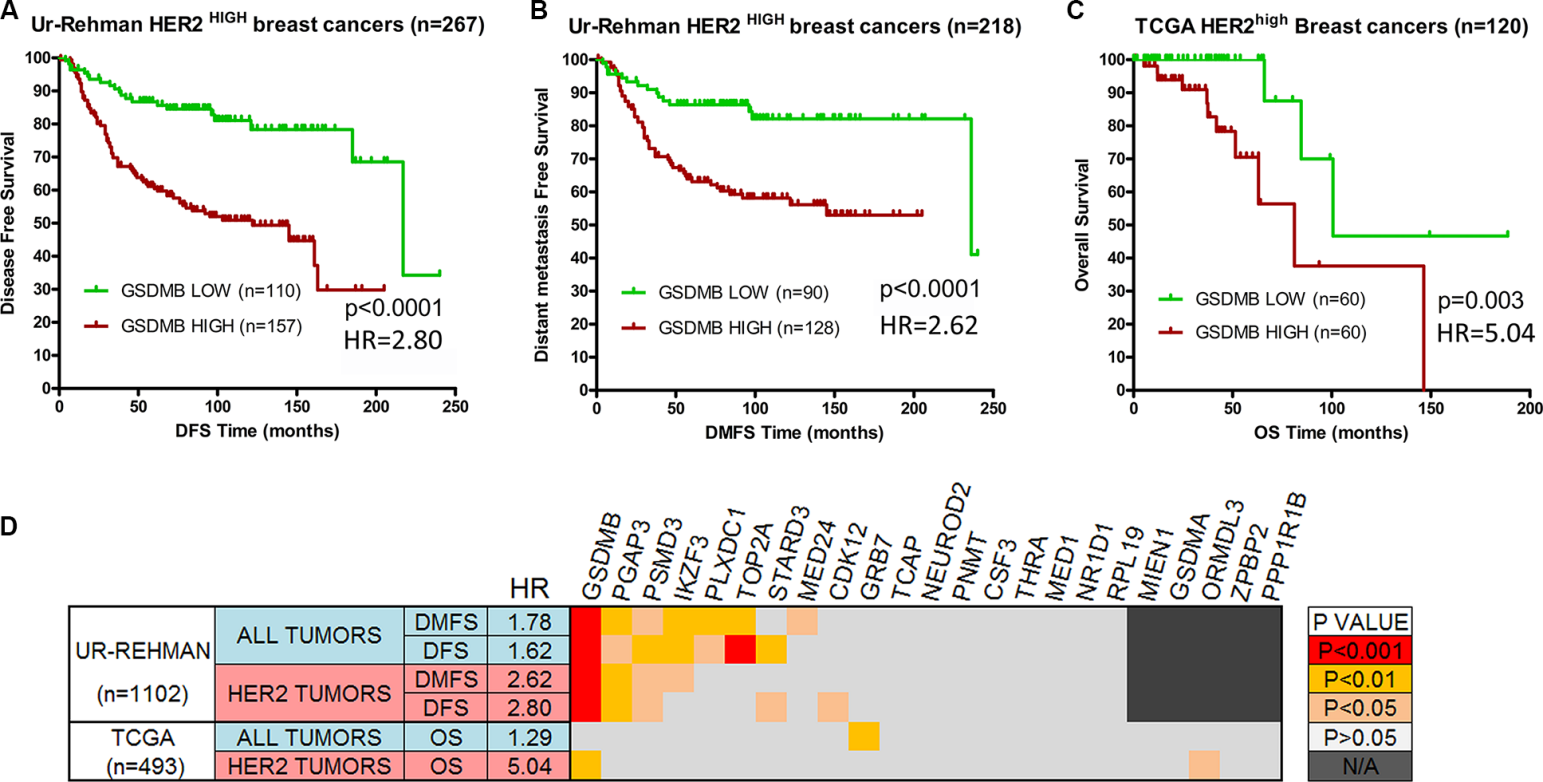
GSDMB over-expression is associated with poor prognosis in ERBB2-high breast cancers Tumor samples with the top 25% expression levels of *GSDMB* gene (“high”) show significantly worse prognosis than the remaining tumors (“low”). (**A**) Disease-free survival and (**B**) Distant metastasis-free survival curves in breast cancers over-expressing (top 25%) *ERBB2* from the Ur-Rehman dataset [35]. (**C**) Overall patient survival curves for *ERBB2*-high tumors in the TCGA dataset [36]. Kaplan-Meier survival curves were generated and differences in survival were assessed by log-rank test (*p* < 0.05 considered statistically significant) using GraphPad PRISM 6.0. (**D**) Survival analyses of 23 genes close to *ERBB2* locus in breast cancers. Tumour samples with the top 25% expression levels of each gene (considered “high”) were compared to the remaining tumors (“low”). Kaplan-Meier survival curves were generated and differences in DFS and DMFS survival were assessed by log-rank Mantel-Cox test using GraphPad PRISM 6.0. Analyses were performed in the Ur-Rehman and the TCGA datasets with all tumours (*n* = 1, 102 and *n* = 493, respectively), and for HER2-high (HER2+) cancers only. The *p* value of each test was color-coded as indicated in the scale on the right. HR: Hazard ratio associated with *GSDMB* gene high expression in each dataset.

Given the above correlations, and the role of GSDMB in promoting an aggressive breast cancer cell phenotype [34], we next assessed if the over-expression of *GSDMB* could also influence the prognosis of HER2-high tumours. As shown in Figure 1, *GSDMB* over-expression in these tumours was significantly associated with poor outcome: shorter disease free survival (DSF) and distant metastasis-free survival (DMFS) in the Ur-Rehman dataset (*p <* 0.001, Figure 1A–1B); as well as overall survival (OS) in the TCGA dataset (*p* < 0.01, Figure 1C). Additionally, the prognosis value of GSDMB in these datasets was not independent of tumor grade. The association with poor prognosis was significantly stronger (higher Hazard Ratios) in HER2-high cancers than in unselected breast cancers (whole dataset, Figure 1D).

Furthermore, we analysed the prognostic value of other 22 genes located near *ERBB2*. As shown in Figure 1D, over-expression of *GSDMB* represented the largest effect relative to poor prognosis (Figure 1D).

Complementarily, to confirm further the association of GSDMB expression with poor prognosis we evaluated an additional series of 58 HER2-positive (by FISH and IHC) breast cancers [38]. Again, higher *GSDMB* expression associates significantly with shorter OS (log-rank *p* = 0.002) (Supplementary Figure 1).

Collectively, consistent results across different datasets demonstrate that high *GSDMB* expression identifies the most aggressive HER2+ breast tumours.

### GSDMB is amplified and over-expressed in HER2-positive breast tumours

**Figure 2:**
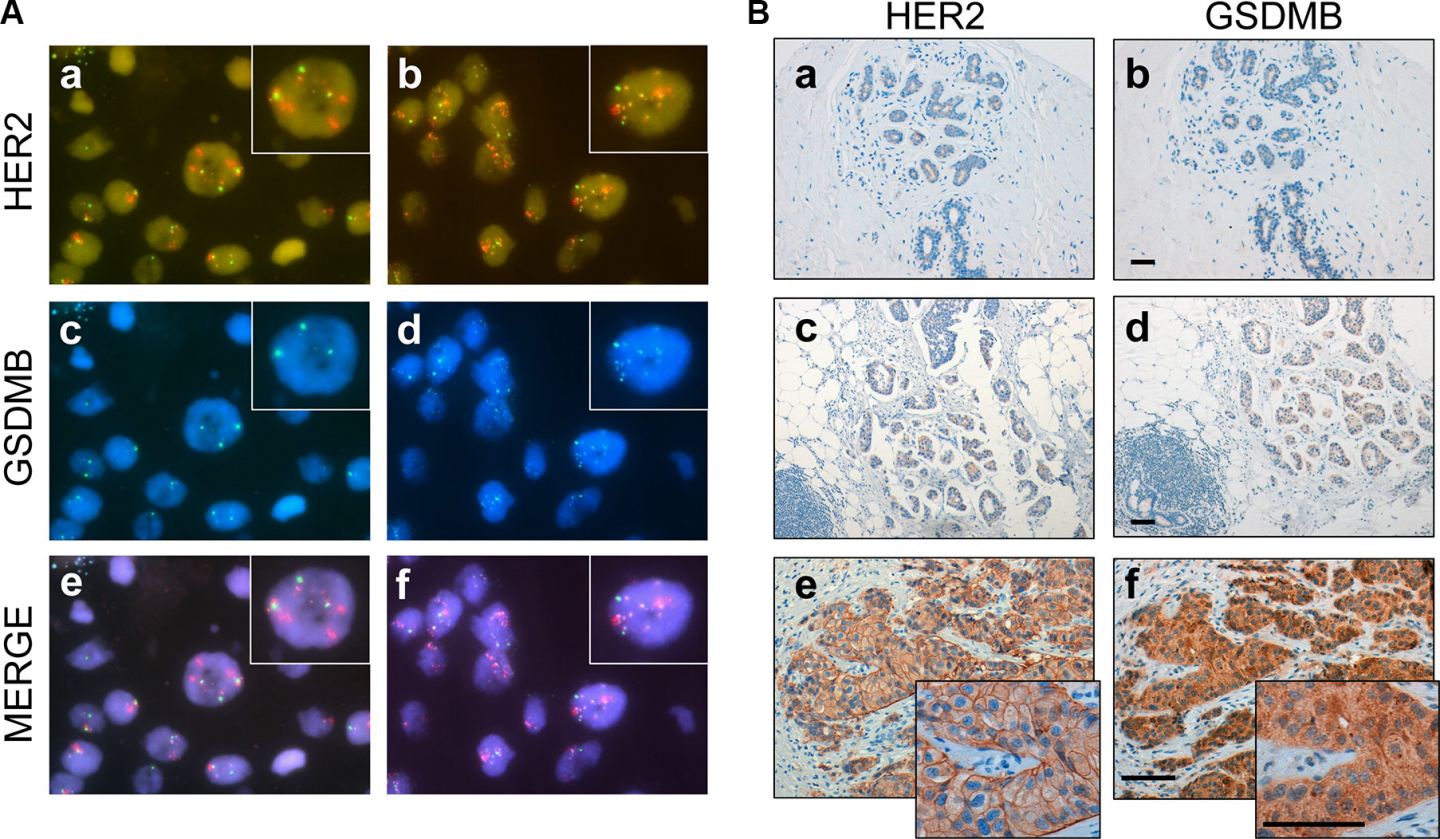
GSDMB gene amplification and protein expression in HER2-positive breast cancer (**A**) Representative breast tumours with positive and negative *HER2* and *GSDMB* amplification. Panels a, c, e: represent an example of breast carcinoma *HER2*-positive (red signal) without *GSDMB* amplification. Panels b, d, f: represent an example of case HER2-positive with *GSDMB* amplification (blue signal). The lower lane shows the merged image (e of a-c and f of b-d panels). Green signals represent CEP7. Magnification x 63x; insets x 120. (**B**) Immunohistochemical analysis of HER2 and GSDMB in normal tissue (panel a, b), and in breast carcinoma cases (panels c–f). Low or undetectable GSDMB staining was observed in normal breast tissue (b) or in lesions with GSDMB gene copy number of < 4 signals/nucleus (d) (considered non-amplified tumours). Intense GSDMB staining in a case with > 10 *GSDMB* copy number (f) considered as GSDMB-positive tumour. Scale bar measures 100 micrometers.

Our *in silico* analyses indicates that *GSDMB* over-expression is associated with the HER2-positive phenotype in breast cancer. To confirm this observation, the copy number status of the *GSDMB* locus was determined by FISH (Figure 2A) in a cohort of 53 breast cancer patients including HER2-positive (*n* = 29) and HER2-negative (*n* = 24) tumours classified according to the *ERBB2* amplification status (“discovery series” in Supplementary Table 2). Thus, *GSDMB* amplification was detected in 21 tumours, and 15 of them (71.4%) were HER2-positive cases, therefore confirming the association between the both amplifications (*p* = 0.034, Table 1, Figure 2A, panel d). In the six cases classified as HER2-negative tumours showing *GSDMB* amplification, the HER2/centromere ratio was 1.9 + 0.24, suggesting the presence of HER2 aneuploidy [39].

**Table 1:** Relationship between GSDMB gene amplification/protein expression and HER2 oncogene in breast cancer “discovery series”

	HER2 amplification: *n (%)	
	Negative	Positive
**GSDMB amplification (*n* = 52)**		
Negative (*n* = 31)	18 (58.1)	13 (41.9)
Positive (*n* = 21)	6 (28.6)	15 (71.4)
		*p* = 0.034
**GSDMB expression (*n* = 52)**		
Negative (*n* = 29)	17 (58.6)	12 (41.4)
Positive (*n* = 23)	7 (30.4)	16 (69.6)
		*p* = 0.040

	GSDMB amplification: *n (%)	
	Negative	Positive
**GSDMB expression (*n* = 52)**		
Negative (*n* = 29)	29 (100)	0
Positive (*n* = 23)	2 (8.7)	21 (91.3)
		*p* < 0.001

* *n* (%), number of analyzed cases and (percentage).

Next, to determine if *GSDMB* amplification leads to over-expression of the corresponding product, an anti-GSDMB monoclonal antibody was developed. The antibody was able to recognize all the isoforms of GSDMB described [34] by Western blot analysis (Supplementary Figure 2 and Materials and Methods) and by immunohistochemistry (IHC). Thus, IHC assays revealed positive expression of GSDMB in 23 of the 52 cases (44.2%; Figure 2B, Table 1). Importantly, this positivity was significantly correlated with *ERBB2* gene amplification (16/23, 69.6%, *p* = 0.040, Table 1) and *GSDMB* amplification (*p* < 0.001; Table 1). In fact, intense GSDMB staining (Figure 2B, panel f) was found in HER2-positive tumours with clear *GSDMB* gene amplification (Figure 2A, panel d, more than 10 signals/nucleus); while undetectable or weak GSDMB staining was detected in normal breast tissue (Figure 2B, panel b) or lesions without *ERBB2* or *GSDMB* amplification (*GSDMB* gene copy number of < 4 signals/nucleus, Figure 2A, panel c). Together, these data demonstrate that GSDMB is over-expressed in a subset (~65%) of HER2-positive tumors, with gene amplification being most likely the mechanism responsible of this alteration.

### GSDMB gene amplification or expression predict poor clinical response and relapse under neoadjuvant settings in HER2-positive breast tumors

The results of the gene expression analyses indicate that *GSDMB* over-expression associates with poor prognosis of HER2-positive breast cancer. Next, we assessed the influence of GSDMB gene amplification and/or expression in the therapeutic response of this cancer subtype. First, the analysis of a series of 28 HER2-positive tumours in the discovery series, treated with the neoadjuvant setting revealed an association between *GSDMB* amplification and the lack of pathologic complete response (pCR) (*p* = 0.001, Table 2a). All of the analysed samples who developed local or distant relapse during the clinical follow-up showed GSDMB gene amplification and/or protein expression, respectively (*p* = 0.030, Table 2a, 2b).

**Table 2:** Relationship between *GSDMB* amplification/expression and clinico-pathological features in HER2-positive breast cancer included in the discovery (*n* = 28) and validation series (*n* = 95)

	Discovery series		Validation series	
(a)	GSDMB amplification: *n (%)		GSDMB amplification: *n (%)	
	Negative	Positive	Negative	Positive
**ER expression**				
Negative	6/9 (66.7)	3/9 (33.3)	11/35 (31.4)	24/35 (68.6)
Positive	7/19 (36.8)	12/19 (63.2)	26/60 (43.3)	34/60 (56.7)
		*p* = 0.142		*p* = 0.176
**PR expression**				
Negative	10/19 (52.6)	9/19 (47.4)	16/49 (32.7)	33/49 (67.3)
Positive	3/9 (33.3)	6/9 (66.6)	19/44 (43.2)	25/44 (56.8)
		*p* = 0.338		*p* = 0.295,
**GSDMB expression**				
Negative	12/12 (100)	0 (0)	29/29 (100)	0 (0)
Positive	1/16 (6.2)	15/16 (93.8)	6/64 (9.4)	58/64 (90.6)
		*p* < 0.001		*p* < 0.001
**pCR** ^†^				
Responders	12/16 (75.0)	4/16 (25.0)	31/58 (53.4)	27/58 (46.6)
Non-responders	1/12 (8.3)	11/12 (91.7)	6/37 (16.2)	31/37 (83.8)
		*p* = 0.001		*p* < 0.001
**Relapse** ^†^				
Negative	12/26 (57.1)	9/26 (42.9)	24/47 (51.1)	23/47 (48.9)
Positive	0 (0)	5/5 (100)	3/19 (15.8)	16/19 (84.2)
		*p* = 0.030		*p* = 0.008

(b)	GSDMB expression: *n (%)		GSDMB expression: *n (%)	
	Negative	Positive	Negative	Positive
**ER expression**				
Negative	6/9 (66.7)	3/9 (79.5)	9/35 (25.7)	26/35 (74.3)
Positive	6/19 (31.6)	13/19 (68.4)	20/58 (34.5)	38/58 (65.5)
		*p* = 0.090		*p* = 0.145
**PR expression**				
Negative	9 (47.4)	10 (52.6)	13/48 (27.1)	35/48 (72.9)
Positive	3 (33.3)	6 (66.7)	14/43 (32.6)	29/43 (67.4)
		*p* = 0.293		*p* = 0.366
**pCR**^†^				
Responders	11 (68.8)	5 (31,3)	25/56 (44.6)	31/56 (55.4)
Non-responders	1 (8.3)	11 (91.7)	4/37 (10.8)	33/37 (89.2)
		*p* = 0.002		*p* < 0.001
**Relapse**^†^				
Negative	11 (52.4)	10 (47.6)	21/46 (45.7)	25/46 (54.3)
Positive	0	5 (100)	2/19 (10.5)	17/19 (89.5)
		*p* = 0.033		*p* = 0.006

The data refers to the available cases for each markers (a) Statistical analysis of GSDMB amplification in breast carcinoma samples included in discovery and validation series. (b) Statistical analysis of GSDMB IHC study in breast carcinoma samples included in discovery and validation series.

* *n* (%), number of analyzed cases and (percentage).

† pCR: pathological complete response when there is no invasive presence of tumour at the breast or ganglia level (< 0.1 mm) and Relapse as local or distant recurrence in HER2-positive tumours.

In order to validate the above results, an independent cohort of 95 HER2-positive breast cancer cases treated with the same neoadjuvant regimens (“validation series”, Supplementary Table 2) was analysed. As expected, 61.1% and 68.8% of the HER2-positive tumours showed *GSDMB* amplification and protein expression, respectively (Supplementary Table 3); in fact, a strong association between both variables was observed (*p* < 0.001; Table 2a). Importantly, the analysis of this series corroborated the association between GSDMB gene amplification/expression and poorer therapy response. Among the 37 non-responder tumours, 31 (83.8%) showed *GSDMB* gene amplification and 33 (89.2%) protein expression (Table 2a–2b). Conversely, few cases without *GSDMB* amplification (16.2%) or protein expression (10.8%) were classified as non-responders (*p* < 0.001, Table 2a–2b). In both series, we did not find a clear association between GSDMB and hormone receptors expression suggesting that GSDMB could be a hormone receptor-independent biomarker.

**Figure 3:**
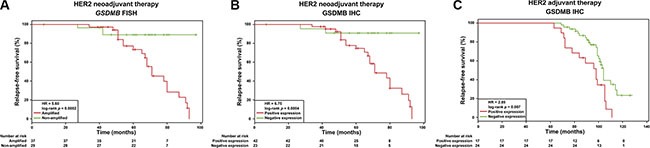
GSDMB gene amplification and/or protein expression associates with relapse in HER2-positive breast cancer samples under neoadjuvant and adjuvant treatment regimens Relapse-free survival curves in HER2-positive breast cancer patients (*n* = 95) treated with neoadjuvant settings in relation to (**A**) *GSDMB* gene amplification (*p* = 0.0002) and (**B**) GSDMB expression scored by immunohistochemistry (IHC, *p* = 0.0004). (**C**) Relapse-free survival curves in HER2- positive breast cancer patients (*n* = 53) treated with adjuvant settings in relation to GSDMB protein expression measured by IHC (*p* = 0.007). Statistical differences, HR and *p*-value, were calculated via log-rank test. Red line: GSDMB amplified and expressed patients, green line: patients without GSDMB amplification and expression.

Next, univariate Cox proportional hazard analyses in the validation series revealed that both variables, *GSDMB* amplification and GSDMB expression, are significantly associated with reduced DFS in the neoadjuvant setting: HR = 5.60, 95% CI 1.66–18.88, long-rank *p* = 0.0002; and HR = 6.75, 95% CI 1.57–28.73, long rank *p* = 0.0004, respectively (Figure 3, Table 3). The average time to relapse for the *GSDMB* amplified and non-amplified cases was 37 ± 11 and 67.3 ± 17 months, respectively (Table 3, Figure 3A). Similarly, GSDMB positive and negative cases identified by IHC presented an average time to relapse of 34.5 ± 14 and 66.2 ± 18 months (Figure 3B). The Kaplan-Meier and log-rank survival analyses further illustrate the significant difference in DFS between GSDMB-positive and GSDMB-negative groups (Figure 3A, 3B). Together, these results indicate that GSDMB expression/amplification, influences both tumour progression and the response to therapy in HER2-positive breast cancer within the neoadjuvant setting.

**Table 3:** Risk of relapse in HER2-positive tumour validation series according to *GSDMB* copy number or GSDMB immunohistochemistry results

Evaluation	Non-relapsed *n, %	Relapsed *n, %	Average time to relapse (months)	HR	95% CI	*p*
**FISH *GSDMB* (*n*, %)**						
**Amplified (37, 56.1%)**	16 (43.2%)	21 (56.8%)	37.0 ± 11	5.60	1.66–18.88	0.0002
**Non-amplified (29, 43.9%)**	26 (89.7%)	3 (10.3%)	67.3 ± 17			
**IHC GSDMB (*n*, %)**						
**Positive (42, 63.6%)**	20 (47.6%)	22 (52.4%)	34.5 ± 14	6.75	1.57 – 28.77	0.0004
**Negative (23, 34.8%)**	21 (91.3%)	2 (8.7%)	66.2 ± 18			

### Clinical association of GSDMB with low therapy response in the adjuvant setting in HER2 -positive breast tumours

To evaluate further the influence of GSDMB expression on the clinical response, a series of 138 breast cases treated with adjuvant therapy were analysed, 53 (41.1%) of these tumours were classified as HER2-positive and were treated following standard adjuvant schedules (Supplementary Table 4). In this series, around 51% of these HER2-positive samples also expressed GSDMB (Table 4). Next, significant associations between GSDMB expression and positive lymph node status (*p* = 0.013) as well as with the existence of distant metastasis (*p* = 0.001) were revealed (Table 4). Univariate Cox proportional hazard analysis confirmed the association with DFS: HR = 2.85, 95% CI 1.28–6.37, log-rank *p* = 0.007 (Table 5). Thus, the average time to relapse for the GSDMB-positive and -negative cases was 91.24 + 14.88 and 101.81 + 9.51 months, respectively. The Kaplan-Meier and log-rank survival analyses further illustrate the difference in DFS between GSDMB-positive and GSDMB-negative cases in the adjuvant setting (*p* = 0.007; Figure 3C).

**Table 4:** Relationship between GSDMB over-expression and clinic pathological features in adjuvant breast cancer series

	GSDMB expression ^†^*n* (%)	
	Negative	Positive
**HER2 amplification (*n* = 127)**		
Negative (*n* = 74)	68 (91.9)	6 (8.1)
Positive (*n* = 53)	26 (49.1)	27 (50.9)
		*p* < 0.001
ER expression (*n* = 43) ^*^		
Negative (*n* = 15)	12 (80.0)	3 (20.0)
Positive (*n* = 28)	11 (39.3)	17 (60.7)
		*p* = 0.012
PR expression (*n* = 49) ^*^		
Negative (*n* = 13)	8 (61.5)	5 (38.5)
Positive (*n* = 36)	17 (47.2)	19 (58.2)
		*p* = 0.489
Lymph node status (*n* = 49) ^*^		
Negative (*n* = 22)	16 (72.7)	6 (27.3)
Positive (*n* = 27)	10 (37.0)	17 (63.0)
		*p* = 0.013
Distant metastasis (*n* = 46) ^*^		
Negative (*n* = 30)	22 (73.3)	8 (26.7)
Positive (*n* = 16)	3 (18.8)	13 (81.2)
		*p* = 0.001

* Only evaluated in HER2-positive breast carcinomas.

† *n* (%), number of analyzed cases and (percentage).

**Table 5:** Risk of relapse in HER2-positive breast tumours treated under adjuvant regimens according to GSDMB immunohistochemistry results

Evaluation	Non-relapsed *n, %	Relapsed *n, %	Average time to relapse (months)	HR	95% CI	*p*
**IHC GSDMB (*n*, %)**						
**Positive (28, 45.2%)**	7 (11.3%)	21 (33.9%)	91.24 ± 14.88	2.85	1.28–6.37	0.007
**Negative (34, 54.8%)**	17 (27.4%)	17 (27.4%)	101.8 ± 9.51			

Overall these results and our previous data firmly establishes GSDMB as a negative prognostic marker in HER2-positive breast cancer patients treated (trastuzumab plus chemotherapy) in both the neoadjuvant and adjuvant settings.

### GSDMB promotes survival to trastuzumab treatment

**Figure 4:**
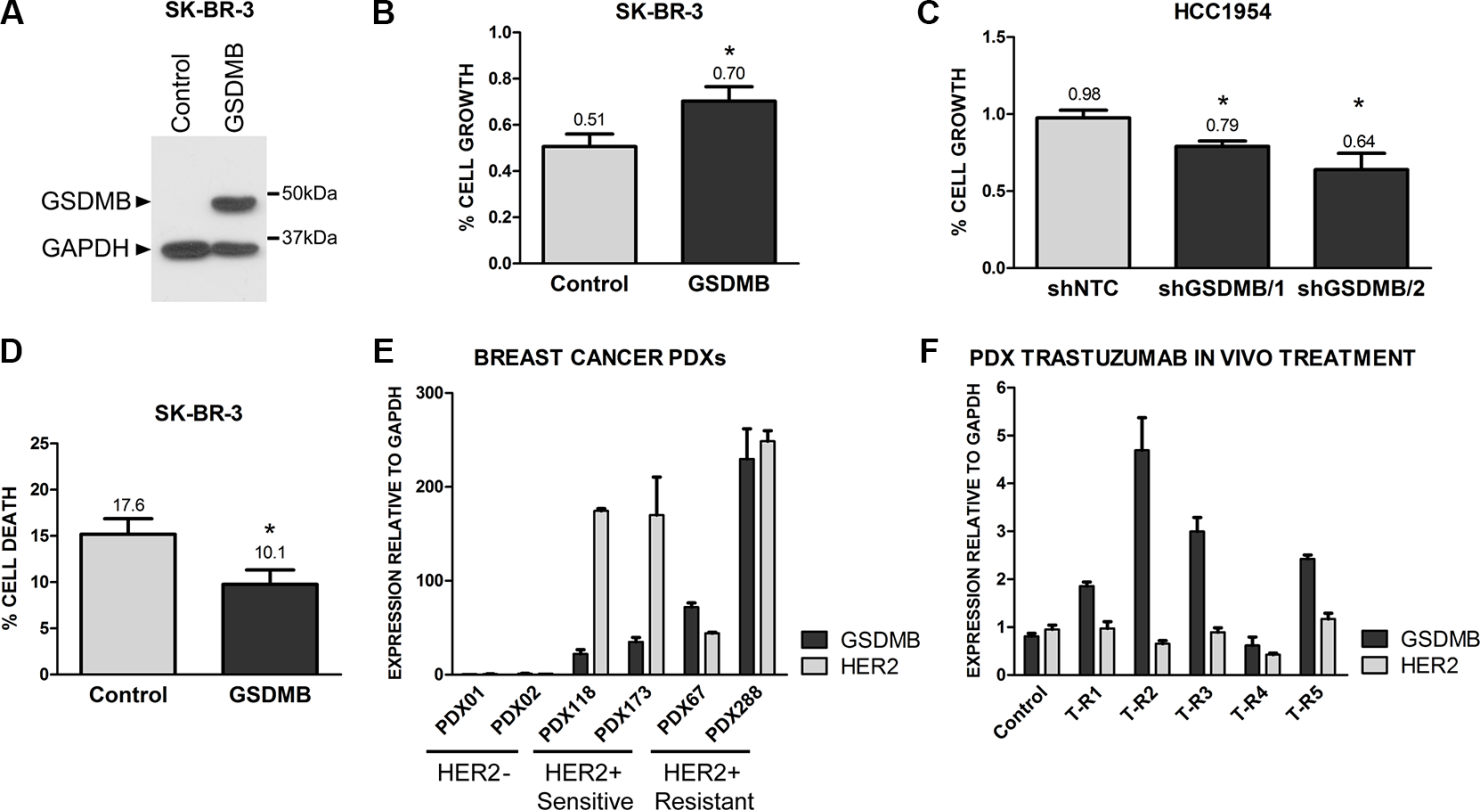
GSDMB expression increases survival to trastuzumab treatment (**A**) Analysis of GSDMB expression by western blot in SKBR3 cells (empty vector, Control) and GSDMB-overexpressing cells (GSDMB). GAPDH was used as a loading control. (**B**) Analysis of cell growth (measured with Alamarblue) in control and GSDMB-overexpressing SKBR3 cells treated with 1 mg/ml trastuzumab for 72 h; (**C**) Reduction of cell growth in control (shNTC) and GSDMB-silenced (two shRNAs) HCC1954 cells treated with 5 mg/ml trastuzumab. (**D**)Analysis of cell death (measured with Annexin V and propidium iodide) in control and GSDMB-overexpressing SKBR3 cells 72 h after trastuzumab treatment. Bars represent mean + SEM of three independent experiments. (**E**) GSDMB and HER2 mRNA expression in Patient Derived Xenografts (PDX) from HER2-negative (PDX01, 02) and HER2-positivie breast carcinomas. PDXs were classified as trastuzumab sensitive (PDX118, 173) or resistant (PDX67, 288) according to *in vivo* patient response [42]. (**F**) GSDMB and HER2 mRNA expression in a trastuzumab-sensitive PDX (PDX118) and its trastuzumab-resistant derived PDXs (T-R1-R5), obtained by chronic *in vivo* treatment with trastuzumab. mRNA values were normalized using GAPDH as housekeeping gene. Bars represent mean + SEM expression of three experiments by quantitative PCR.

Finally, to assess if GSDMB expression was functionally involved in promoting survival to anti-HER2 therapy we performed several complementary experimental approaches. First, we evaluated the potential role of GSDMB expression on trastuzumab response in HCC1954 cells in which endogenous GSDMB has been stably silenced by shRNAs [34]. This model is an example of trastuzumab-resistant HER2+ human breast cancer cell line [40]. Additionally, we generated GSDMB exogenously expressing cells using SKBR3 (Figure 4A), which are trastuzumab-responsive cells [40]. In both cell models, trastuzumab inhibition of cell growth (measured by Alamar Blue method) was significantly higher (~20%) in cells with low GSDMB expression (Figure 4B–4C). Moreover, GSDMB significantly increased cell survival to trastuzumab treatment, as demonstrated by the reduction in apoptosis in GSDMB-overexpressing SKBR3 cells compared to control cells (Figure 4D). Given that HCC1954 are intrinsically very resistant to trastuzumab (5 mg/ml) we could not test the effect on apoptosis in this cell line.

Second, to demonstrate the association of GSDMB with resistance *in vivo* we utilized six breast cancer Patient Derived Xenografts (PDX), a more clinically relevant model [41]. We used four HER2-positive breast cancer PDXs, two sensitive and two primary resistant to trastuzumab [42] and data not shown) and two HER2-negative PDX as control (Figure 4E). The PDXs that did not respond to trastuzumab *in vivo* exhibited higher mRNA (Figure 4E) and protein levels (Supplementary Figure 3) of GSDMB than trastuzumab-sensitive patients and HER2-negative PDXs. Next, one of the trastuzumab-sensitive PDX (PDX118) was treated *in vivo* (biweekly; 1 mg/ml) with trastuzumab to induce an acquired drug resistance [43]. In most of the resistant PDXs originated from PDX118, we observed a significant increase in GSDMB (but not HER2) levels, thus corroborating that its expression is specifically associated with *in vivo* resistance to trastuzumab (Figure 4F).

Altogether, these data demonstrate that GSDMB overexpression is functionally involved in promoting resistance to the anti-HER2 inmunotherapy trastuzumab, and this may explain its association with adverse clinical behaviour in HER2+ cancers.

## DISCUSSION

Despite significant clinical benefits to trastuzumab in HER2-positive breast tumors, not all patients respond well to this therapy, and therefore experience disease recurrence and progression [44, 45], within a few months of therapy (median of 7.4 months) [8, 13]. In this scenario, the mechanisms of resistance to HER2-targeted therapy are not completely understood [46], and this limitation includes the unknown role of 17q12-q21 genes linked to *ERBB2*.

Our study demonstrates that gasdermin B (*GSDMB*), mapped 175 kilo bases distal to *ERBB2*, is amplified and over-expressed in a subset (~60%) of HER2-positive breast tumours. Importantly, GSDMB gene and protein over-expression is significantly associated with poor clinical outcome, in terms of disease progression, relapse and response after neoadjuvant therapy (Table 2), as well as lymph node positivity and the development of distant metastases in the adjuvant context (Table 4) independently of hormone receptors status. Notably, the estimated effect in the neoadjuvant setting is relatively high (HR > 5) and, hence, the average time to relapse is almost halved in cases with *GSDMB* amplification or GSDMB over-expression.

GSDMB belongs to the gasdermin family of proteins [47]. Four gasdermin (*GSDM*) gene members have been identified in humans (GSDMA-D) [48]. GSDM genes have been involved in secretion, proliferation and differentiation in various tissues [48], although their major role seems to be the regulation of diverse types of cell death. In fact, GSDMD has been reported to be a substrate of caspase 11, essential for the activation of pyroptosis as anti-bacterial innate immune defence [49–52] and GSDMA promotes cell death through autophagy and mitochondrial damage [49, 50]. The cell-death promoting function of GSDMA, C and D, depends on the cleavage of their N-terminal portion [51]. Interestingly, GSDMB does not induce cell death, and its C-terminal region is able to reduce partially the cell death induced by GSDMA [50]. Consistently, our data in different breast cancer cell lines models as well as in tumor Patient Derived Xenografts (PDXs) indicate that GSDMB promotes cell survival to trastuzumab anti-HER2 immunotherapy. Given that GSDMB alone does not affect cell growth [34], these results point out that GSDMB might be a negative regulator of cell death/survival under certain conditions (anti-HER2 therapy). Additionally, we have previously shown that GSDMB over-expression in a breast cancer cell line increased tumorigenic and invasive behaviour [34]. Therefore, GSDMB is functionally involved in promoting aggressive tumour behaviour and reduced clinical response to anti-HER2 therapies. It would be of interest in the future to decipher the exact molecular mechanisms by which GSDMB mediates these effects.

In conclusion, our study has identified GSDMB as a relevant biomarker of poor prognosis that is also able to predict non-response to current standard therapy for HER2-positive breast cancer. Since GSDMB expression promotes cell survival to anti-HER2 treatment, the analysis of *GSDMB* gene status and/or GSDMB expression may lead to more precise molecular classification of this cancer subtype and, in turn, help to identify cases that may not benefit from the current therapies.

## MATERIALS AND METHODS

### Tumour samples

A total of 286 paraffin-embedded ductal breast carcinoma included in this study was collected between 2003 and 2014 at the Vall d'Hebron Hospital (Barcelona), Virgen del Rocío Hospital (Seville, Spain) and the MD Anderson Cancer Centre (Madrid). This study was approved by the local ethical committee from each institution, and a complete written informed consent was obtained from all patients. Briefly, this study was performed in two independent data samples treated under neoadjuvant treatment: discovery series, which included 29 HER2 positive and 24 HER2 negative breast carcinomas, and validation series composed by 95 HER2 positive breast carcinomas (Supplementary Tables 2 and 3). The median age of patients and tumour size was 53.2 ± 14.9 years and 2.23 ± 1.45 cm, respectively. Additionally, we studied 138 high-grade ductal breast tumours (52 HER2-positive and 86 HER2-negative samples) all of them treated with adjuvant schedules (Supplementary Table 4). The mean age of patient and tumour size at surgery was 59.6 ± 9,1 years and 1.69 ± 0.42 cm, respectively. HER2 positivity was considered when the tumours showed gene amplification. All cases were treated with trastuzumab plus chemotherapy (based on a combination of anthracyclines and taxanes). Hormone therapy was added in those cases with positive hormone receptor expression. The clinical response of HER2-positive cases in the neoadjuvant setting was evaluated using the pathological complete response criteria (pCR). The pCR was assessed at the breast parenchyma and at the ganglia level. A patient will be deemed to be in pCR when there is no invasive presence of tumour at the breast or ganglia level.

### Immunohistochemistry (IHC) and Fluorescence *in situ* hybridization (FISH) analysis

HER2, ER (oestrogen receptor) and PR (progesterone receptor) were analysed by IHC in TMAs and in a complete section samples from PDXs, according to standard methods as previously published [40]. GSDMB monoclonal antibody was generated as described in Supplementary Information, and GSDMB expression was classified as positive when more than 10% of tumour cells showed positive cytoplasmic staining. All studied tumour sections also included normal breast tissue as an internal control. In negative controls, the primary antibodies were omitted. FISH study was performed as previously described [53]. Briefly, the analysis was carried out simultaneously using red-labelled *ERBB2*, green-labelled chromosome enumeration probe (CEP17) as control (PathVysion Kit, Abbott Molecular), and aqua-labelled *GSDMB*. The *GSDMB* probe was generated from DNA isolated from the bacterial artificial clone (BAC) RP11–387H17 obtained from the BACPAC Resource Centre (BPRC) at the Children's Hospital Oakland Research Institute (CA, USA) and labelled by nick translation. Fluorescence signals were scored in each sample by counting the number of single copy genes and control probe signals in an average of 180 (100–210) well-defined nuclei. The ASCO CAP 2007 criteria for *ERBB2* gene amplification [39] were used to evaluate the status of *GSDMB* gene. Increased gene copy number and amplification were defined as the presence of 4–6 and > 6 signals, respectively. The GSDMB/CEP17 ratio used to delimit amplification was of ≥ 2.2 [39].

### *GSDMB* expression analysis in breast cancer microarray datasets

To evaluate the expression of *GSDMB* in breast tumours, several independent datasets were analyzed [35, 36, 38] Microarray and clinical data were obtained from the ICR database (www.rock.icr.ac.uk). For each gene, normalized expression was categorized as “high” when it was above the third quartile (top 25% expression) of all tumour samples; otherwise, it was categorized as “low”. Kaplan-Meier estimates of *GSDMB* expression (assessed separately for each stratum) were plotted and compared to overall survival (OS), disease-free survival (DFS) and metastasis-free survival (MFS) curves using log-rank and Chi-square tests.

### Cell lines and Patient Derived Xenografts (PDXs)

For the study of the role of GSDMB in response to trastuzumab two well-studied HER2-positive breast cancer cell lines [40], HCC1954 and SKBR3, were used. Both cell lines were obtained from the American Type Cell Culture (ATCC). Cells were grown as monolayer cultures at 37°C in an atmosphere with 5% CO2 and authenticated by STR-profiling according to ATCC guidelines. For the analysis of GSDMB over-expression, SKBR3 cells were stably transfected using Lipofectamine (Invitrogen) with pEZ-M61-GSDMB (Genecopoeia) or the empty vector. Cells containing the plasmids were selected with G418 (0,8 mg/ml) treatment. GSDMB-silenced HCC1954 cells were obtained as previously reported [34]. HER2-positive and trastuzumab resistant Patient Derived Xenografts (PDXs) were previously described in [42, 43]. HER2-negative PDXs were obtained from primary triple-negative breast tumours after engrafting orthotopically in the mammary fat of female athymic (nu/nu) mice (Harlan^™^). The patients provided written informed consent nd the study was approved by IDIBELL Ethics committee. All ortho-xenograft models were established for > 3 passes. Their triple negative status was confirmed following standard inmmunohistochemical assays.

### Cell-based assays

Trastuzumab anti-proliferative effects were measured in 2 × 10^3^ cells plated on 96-well-plates using alamarBlue assay (Thermo Scientific) according to the manufacturer's protocol. HCC1953 and SKBR3 cells were daily treated up to 72 h with 5 mg/ml and 1 mg/ml trastuzumab (Roche Diagnostics GmbH, Penzberg, Germany), respectively, or were left untreated. For detection of the death rate, SKBR3 cells were pre-treated in 0,1% FBS-containing medium during 24 h, and then (50 × 10^3^.000 cells) cells were cultured in 6-well plates and treated with or without trastuzumab (1 mg/mL dissolved in medium) for 72 hours. Cell death was measured by staining with Annexin V and propidium iodide (PI) using PE-Apoptosis Detection kit (Immunostep) according to the manufacturer's instructions, and analyzed on a FACSCANTO II flow cytometer (BD Biosciences). Three independent experiments were performed for Alamar blue and Cell death measurements.

### Gene expression assays

Gene expression was performed after extraction of total RNA from PDXs using RNA miniprep system, ReliaPrep^TM^ FFPE total (Promega) following manufacturer recommendations. cDNA was obtained from 1 μg of total RNA using random primers and M-MLV reverse transcriptase (Amresco) as manufacturer recommendations. GSDMB, HER2, and GAPDH gene expression levels were measured by quantitative real time RT-PCR (qRT-PCR) using pre-designed TaqMan probes (Thermofisher) and PerfeCTa FastmixII (Quanta BioSciences, Inc), on an iQ5 iCycler Realtime PCR Detection System (BioRad), according to the manufacturer's recommendations. All qRT-PCRs were performed in triplicate. Relative GSDMB and HER2 expression was normalized to GAPDH.

### Statistical analysis

The χ^2^ or Fisher's exact tests were used to test associations between categorical variables. All tests were two-tailed and 95% confidence intervals (CIs) were used. Values of *p* < 0.05 were considered statistically significant. Hazard ratios (HRs) and 95% CIs were estimated from univariate or multivariate Cox proportional hazards models. Disease-free survival was defined as the time from the date of diagnosis to the date of recurrence or development of novel distant metastasis. Survival curves were generated using the Kaplan-Meier method. The *p*-values are based on the Wald test for Cox proportional hazards models, and the log-rank test for Kaplan-Meier analysis. Statistical analysis were performed in R and using the Survival package and SPSS Statistics 17.0 (SPSS Inc., Chicago, IL).

## SUPPLEMENTARY MATERIALS FIGURES AND TABLES



## References

[1] Ross JS, Slodkowska EA, Symmans WF, Pusztai L, Ravdin PM, Hortobagyi GN (2009). The HER-2 receptor and breast cancer: ten years of targeted anti-HER-2 therapy and personalized medicine. Oncologist.

[2] Dawood S, Broglio K, Buzdar AU, Hortobagyi GN, Giordano SH (2010). Prognosis of women with metastatic breast cancer by HER2 status and trastuzumab treatment: an institutional-based review. J Clin Oncol.

[3] Eroles P, Bosch A, Pérez-Fidalgo JA, Lluch A (2012). Molecular biology in breast cancer: Intrinsic subtypes and signaling pathways. Cancer Treat Rev.

[4] Slamon DJ, Leyland-Jones B, Shak S, Fuchs H, Paton V, Bajamonde A, Fleming T, Eiermann W, Wolter J, Pegram M, Baselga J, Norton L (2001). Use of chemotherapy plus a monoclonal antibody against HER2 for metastatic breast cancer that overexpresses HER2. N Engl J Med.

[5] Gianni L, Dafni U, Gelber RD, Azambuja E, Muehlbauer S, Goldhirsch A, Untch M, Smith I, Baselga J, Jackisch C, Cameron D, Mano M, Pedrini JL, Herceptin Adjuvant (HERA) Trial Study Team (2011). Treatment with trastuzumab for 1 year after adjuvant chemotherapy in patients with HER2-positive early breast cancer: a 4-year follow-up of a randomised controlled trial. Lancet Oncol.

[6] Krop IE, Lin NU, Blackwell K, Guardino E, Huober J, Lu M, Miles D, Samant M, Welslau M, Diéras V (2015). Trastuzumab emtansine (T-DM1) versus lapatinib plus capecitabine in patients with HER2-positive metastatic breast cancer and central nervous system metastases: a retrospective, exploratory analysis in EMILIA. Ann Oncol.

[7] Montemurro F, Valabrega G, Aglietta M (2007). Lapatinib: A dual inhibitor of EGFR and HER2 tyrosine kinase activity. Expert Opin Biol Ther.

[8] Slamon D, Eiermann W, Robert N, Pienkowski T, Martin M, Press M, Mackey J, Glaspy J, Chan A, Pawlicki M, Pinter T, Valero V, Liu MC, Breast Cancer International Research Group (2011). Adjuvant trastuzumab in HER2-positive breast cancer. N Engl J Med.

[9] Verma S, Miles D, Gianni L, Krop IE, Welslau M, Baselga J, Pegram M, Oh DY, Diéras V, Guardino E, Fang L, Lu MW, Olsen S (2012). Trastuzumab emtansine for HER2-positive advanced breast cancer. N Engl J Med.

[10] Swain SM, Kim SB, Cortés J, Ro J, Semiglazov V, Campone M, Ciruelos E, Ferrero JM, Schneeweiss A, Knott A, Clark E, Ross G, Benyunes MC (2013). Pertuzumab, trastuzumab, and docetaxel for HER2-positive metastatic breast cancer (CLEOPATRA study): overall survival results from a randomised, double-blind, placebo-controlled, phase 3 study. Lancet Oncol.

[11] Guarneri V, Dieci MV, Barbieri E, Piacentini F, Omarini C, Ficarra G, Bettelli S, Conte PF (2013). Anti-HER2 neoadjuvant and adjuvant therapies in HER2 positive breast cancer. Ann Oncol.

[12] Nielsen DL, Kümler I, Palshof JA, Andersson M (2013). Efficacy of HER2-targeted therapy in metastatic breast cancer. Monoclonal antibodies and tyrosine kinase inhibitors. Breast.

[13] de Azambuja E, Holmes AP, Piccart-Gebhart M, Holmes E, Di Cosimo S, Swaby RF, Untch M, Jackisch C, Lang I, Smith I, Boyle F, Xu B, Barrios CH (2014). Lapatinib with trastuzumab for HER2-positive early breast cancer (NeoALTTO): survival outcomes of a randomised, open-label, multicentre, phase 3 trial and their association with pathological complete response. Lancet Oncol.

[14] Sahlberg KK, Hongisto V, Edgren H, Mäkelä R, Hellström K, Due EU, Moen Vollan HK, Sahlberg N, Wolf M, Børresen-Dale AL, Perälä M, Kallioniemi O (2013). The HER2 amplicon includes several genes required for the growth and survival of HER2 positive breast cancer cells. Mol Oncol.

[15] Gianni L, Eiermann W, Semiglazov V, Lluch A, Tjulandin S, Zambetti M, Moliterni A, Vazquez F, Byakhov MJ, Lichinitser M, Climent MA, Ciruelos E, Ojeda B (2014). Neoadjuvant and adjuvant trastuzumab in patients with HER2-positive locally advanced breast cancer (NOAH): follow-up of a randomised controlled superiority trial with a parallel HER2-negative cohort. Lancet Oncol.

[16] Perez EA, Romond EH, Suman VJ, Jeong JH, Sledge G, Geyer CE, Martino S, Rastogi P, Gralow J, Swain SM, Winer EP, Colon-Otero G, Davidson NE (2014). Trastuzumab plus adjuvant chemotherapy for human epidermal growth factor receptor 2-positive breast cancer: planned joint analysis of overall survival from NSABP B-31 and NCCTG N9831. J Clin Oncol.

[17] Arriola E, Marchio C, Tan DS, Drury SC, Lambros MB, Natrajan R, Rodriguez-Pinilla SM, Mackay A, Tamber N, Fenwick K, Jones C, Dowsett M, Ashworth A (2008). Genomic analysis of the HER2/TOP2A amplicon in breast cancer and breast cancer cell lines. Lab Invest.

[18] Kao J, Pollack JR (2006). RNA interference-based functional dissection of the 17q12 amplicon in breast cancer reveals contribution of coamplified genes. Genes Chromosomes Cancer.

[19] Kauraniemi P, Kuukasjärvi T, Sauter G, Kallioniemi A (2003). Amplification of a 280-kilobase core region at the ERBB2 locus leads to activation of two hypothetical proteins in breast cancer. Am J Pathol.

[20] Staaf J, Jönsson G, Ringnér M, Vallon-Christersson J, Grabau D, Arason A, Gunnarsson H, Agnarsson BA, Malmström PO, Johannsson OT, Loman N, Barkardottir RB, Borg A (2010). High-resolution genomic and expression analyses of copy number alterations in HER2-amplified breast cancer. Breast Cancer Res.

[21] Jacot W, Fiche M, Zaman K, Wolfer A, Lamy PJ (2013). The HER2 amplicon in breast cancer: Topoisomerase IIA and beyond. Biochim Biophys Acta.

[22] Hongisto V, Aure MR, Mäkelä R, Sahlberg KK (2014). The HER2 amplicon includes several genes required for the growth and survival of HER2 positive breast cancer cells- A data description. Genom Data.

[23] Nadler Y, González AM, Camp RL, Rimm DL, Kluger HM, Kluger Y (2010). Growth factor receptor-bound protein-7 (Grb7) as a prognostic marker and therapeutic target in breast cancer. Ann Oncol.

[24] Press MF, Sauter G, Buyse M, Bernstein L, Guzman R, Santiago A, Villalobos IE, Eiermann W, Pienkowski T, Martin M, Robert N, Crown J, Bee V (2011). Alteration of topoisomerase II-alpha gene in human breast cancer: association with responsiveness to anthracycline-based chemotherapy. J Clin Oncol.

[25] Arriola E, Rodriguez-Pinilla SM, Lambros MB, Jones RL, James M, Savage K, Smith IE, Dowsett M, Reis-Filho JS (2007). Topoisomerase II alpha amplification may predict benefit from adjuvant anthracyclines in HER2 positive early breast cancer. Breast Cancer Res Treat.

[26] Katoh M, Katoh M (2004). Evolutionary recombination hotspot around GSDML-GSDM locus is closely linked to the oncogenomic recombination hotspot around the PPP1R1B-ERBB2-GRB7 amplicon. Int J Oncol.

[27] Katoh M, Katoh M (2004). Identification and characterization of human DFNA5L, mouse Dfna5l, and rat Dfna5l genes *in silico*. Int J Oncol.

[28] Watabe K, Ito A, Asada H, Endo Y, Kobayashi T, Nakamoto K, Itami S, Takao S, Shinomura Y, Aikou T, Yoshikawa K, Matsuzawa Y, Kitamura Y (2001). Structure, expression and chromosome mapping of MLZE, a novel gene which is preferentially expressed in metastatic melanoma cells. Jpn J Cancer Res.

[29] Saeki N, Kuwahara Y, Sasaki H, Satoh H, Shiroishi T (2000). Gasdermin (Gsdm) localizing to mouse chromosome 11 is predominantly expressed in upper gastrointestinal tract but significantly suppressed in human gastric cancer cells. Mamm Genome.

[30] Carl-McGrath S, Schneider-Stock R, Ebert M, Röcken C (2008). Differential expression and localisation of gasdermin-like (GSDML), a novel member of the cancer-associated GSDMDC protein family, in neoplastic and non-neoplastic gastric, hepatic, and colon tissues. Pathology.

[31] Sun Q, Yang J, Xing G, Sun Q, Zhang L, He F (2008). Expression of GSDML Associates with Tumor Progression in Uterine Cervix Cancer. Transl Oncol.

[32] Van Laer L, Huizing EH, Verstreken M, van Zuijlen D, Wauters JG, Bossuyt PJ, Van de Heyning P, McGuirt WT, Smith RJ, Willems PJ, Legan PK, Richardson GP, Van Camp G (1998). Nonsyndromic hearing impairment is associated with a mutation in DFNA5. Nat Genet.

[33] Wu H, Romieu I, Sienra-Monge JJ, Li H, del Rio-Navarro BE, London SJ (2009). Genetic variation in ORM1-like 3 (ORMDL3) and gasdermin-like (GSDML) and childhood asthma. Allergy.

[34] Hergueta-Redondo M, Sarrió D, Molina-Crespo Á, Megias D, Mota A1, Rojo-Sebastian A, García-Sanz P, Morales S, Abril S, Cano A, Peinado H, Moreno-Bueno G (2014). Gasdermin-B promotes invasion and metastasis in breast cancer cells. PLoS One.

[35] Ur-Rehman S, Gao Q, Mitsopoulos C, Zvelebil M (2013). ROCK: a resource for integrative breast cancer data analysis. Breast Cancer Res Treat.

[36] Cancer Genome Atlas Network (2012). Comprehensive molecular portraits of human breast tumours. Nature.

[37] Ebbert MT, Bastien RR, Boucher KM, Martín M, Carrasco E, Caballero R, Stijleman IJ, Bernard PS, Facelli JC (2011). Characterization of uncertainty in the classification of multivariate assays: application to PAM50 centroid-based genomic predictors for breast cancer treatment plans. J Clin Bioinforma.

[38] Staaf J, Ringnér M, Vallon-Christersson J, Jönsson G, Bendahl PO, Holm K, Arason A, Gunnarsson H, Hegardt C, Agnarsson BA, Luts L, Grabau D, Fernö M (2010). Identification of subtypes in human epidermal growth factor receptor 2—positive breast cancer reveals a gene signature prognostic of outcome. J Clin Oncol.

[39] Wolff AC, Hammond ME, Schwartz JN, Hagerty KL, Allred DC, Cote RJ, Dowsett M, Fitzgibbons PL, Hanna WM, Langer A, McShane LM, Paik S, Pegram MD (2007). American Society of Clinical Oncology/College of American Pathologists guideline recommendations for human epidermal growth factor receptor 2 testing in breast cancer. Arch Pathol Lab Med.

[40] Henjes F, Bender C, von der Heyde S, Braun L, Mannsperger H A, Schmidt C, Wiemann S, Hasmann M, Aulmann S, Beissbarth T, Korf U (2012). Strong EGFR signaling in cell line models of ERBB2-amplified breast cancer attenuates response towards ERBB2-targeting drugs. Oncogenesis.

[41] Whittle JR, Lewis MT, Lindeman GJ, Visvader JE (2015). Patient-derived xenograft models of breast cancer and their predictive power. Breast Cancer Res.

[42] Vicario R, Peg V, Morancho B, Zacarias-Fluck M, Zhang J, Martínez-Barriocanal Á, Navarro Jiménez A, Aura C, Burgues O, Lluch A, Cortés J, Nuciforo P, Rubio IT (2015). Patterns of HER2 Gene Amplification and Response to Anti-HER2 Therapies. PLoS One.

[43] Parra-Palau J Ll, Morancho B, Peg P, Scaltriti M, Vicario R, Zacarias-Fluck M, Pedersen K, Pandiella A, Nuciforo P, Serra V, Cortés J, Baselga JM, Perou CM (2014). Effect of p95HER2/611CTF on the Response to Trastuzumab and Chemotherapy. J Natl Cancer Inst.

[44] Ballinger TJ, Sanders ME, Abramson VG (2015). Current HER2 Testing Recommendations and Clinical Relevance as a Predictor of Response to Targeted Therapy. Clin Breast Cancer.

[45] Crozier JA, Swaika A, Moreno-Aspitia A (2014). Adjuvant chemotherapy in breast cancer: To use or not to use, the anthracyclines. World J Clin Oncol.

[46] Hubalek M, Brunner C, Mattha K, Marth C (2010). Resistance to HER2-targeted therapy: mechanisms of trastuzumab resistance and possible strategies to overcome unresponsiveness to treatment. Wien Med Wochenschr.

[47] Fujii T, Tamura M, Tanaka S, Kato Y, Yamamoto H, Mizushina Y, Shiroishi T (2008). Gasdermin D (Gsdmd) is dispensable for mouse intestinal epithelium development. Genesis.

[48] Saeki N, Sasaki H, Carrasco J, Mota M (2012). Gasdermin Superfamily: a novel gene family functioning in epithelial cells. Endothelium and Epithelium: composition, functions and pathology., Inc.

[49] Kayagaki N, Stowe IB, Lee BL, O'Rourke K, Anderson K, Warming S, Cuellar T, Haley B, Roose-Girma M, Phung QT, Liu PS, Lill JR, Li H (2015). Caspase-11 cleaves gasdermin D for non-canonical inflammasome signalling. Nature.

[50] Shi J, Zhao Y, Wang K, Shi X, Wang Y, Huang H, Zhuang Y, Cai T, Wang F, Shao F (2015). Cleavage of GSDMD by inflammatory caspases determines pyroptotic cell death. Nature.

[51] He WT, Wan H, Hu L, Chen P, Wang X, Huang Z, Yang ZH, Zhong CQ, Han J (2015). Gasdermin D is an executor of pyroptosis and required for interleukin-1β secretion. Cell Res.

[52] Man SM, Kanneganti TD (2015). Gasdermin D: the long-awaited executioner of pyroptosis. Cell Res.

[53] Martínez-Ramírez A, Urioste M, Melchor L, Blesa D, Valle L, de Andrés SA, Kok K, Calasanz MJ, Cigudosa JC, Benítez J (2005). Analysis of myelodysplastic syndromes with complex karyotypes by high-resolution comparative genomic hybridization and subtelomeric CGH array. Genes Chromosomes Cancer.

