# Supplementary material for: Gasdermin B expression predicts poor clinical outcome in HER2-positive breast cancer
Source: Oncotarget. 2016 Jul 22;7(35):56295–308. doi: 10.18632/oncotarget.10787 (PMC5302915; doi:10.18632/oncotarget.10787)
Supplement: Supplementary file 1 [file oncotarget-07-56295-s001.pdf]

Gasdermin B expression predicts poor clinical outcome in HER2-positive breast cancer

Supplementary Materials

SUPPLEMENTARY METHODS

Generation of GSDMB antibody

Two BALB/c mice were intra-peritoneal injected (three times at 14-day intervals) with 100 µg of a peptide comprising amino acids 208–406 of the C-terminal region of a His-GSDMB fusion protein and complete Freund›s adjuvant (Difco). A 150 µg booster of the recombinant His-GSDMB protein was injected intra-peritoneally, and fused three days later by conventional methods. The cell fusion partner was the NS-1 myeloma cell line (P3/NS1/1-Ag4-1). Hybridoma supernatants were screened by ELISA and by western blot using HEK-293T cells transfected with pCDNA3-HA-Gasdemin B plasmid. The monoclonal antibody (mAb; clone GAS120C, isotype IgG2b) was cloned by limiting dilution. Antibody purification was performed with a Hi-Trap Protein G column (GE Healthcare, UK). Animal experiments were performed under the experimental protocol approved by the Institutional Committee for Care and Use of Animals (CEUCA 001/002). To confirm that the mAb recognized the human GSDMB protein, the HEK-293T cell line was transiently transfected with each of the GSDMB isoforms previously characterized [34]. Briefly, the HEK-293T cell line was transiently transfected with Lipofectamine 2000 (InvitroGen) and 4 µg of pEZ-M61 expression vectors (GeneCopoeia) containing the cDNA of each of the described GSDMB isoforms (isoform 1, NM_001042471.1; isoform 2, NM_018530.2; isoform 3 NM_001165958.1; and isoform 4, NM_001165959.1). After 48 h cells were lysed in RIPA buffer and 40 µg of total proteins were run in 15% SDS-PAGE Gels. Membranes were incubated overnight with the mouse monoclonal anti-GSDMB antibody described above (1:250 in 5% milk) and then with anti-mouse IgGs-HRP (1:5000; 1 h). Four bands matching the predicted size of each GSDMB isoform were detected. No additional (unspecific) bands were observed.

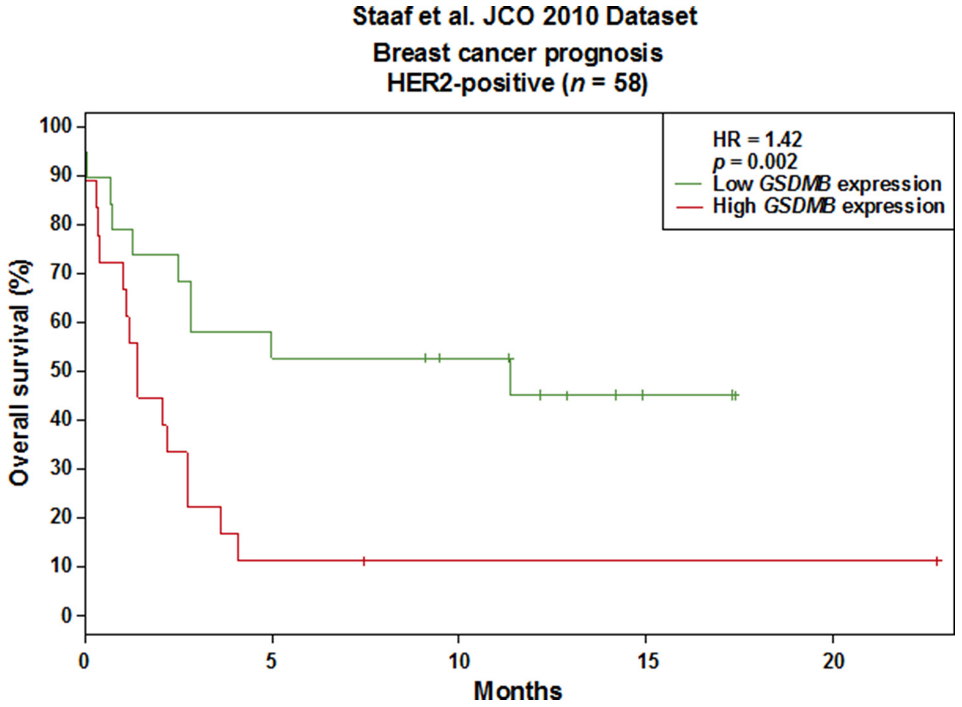

Supplementary Figure S1: *GSDMB* over-expression is associated with poor overall survival in HER2-positive breast cancers. Tumour samples with the top 25% mRNA expression levels of *GSDMB* gene ("high", red) show significantly worse prognosis than the remaining tumors ("low", green). Overall Survival curve in HER2-positive breast cancers patients from the Staaf dataset [38]. C Statistical differences, HR and *p*-value, were calculated via log-rank test.

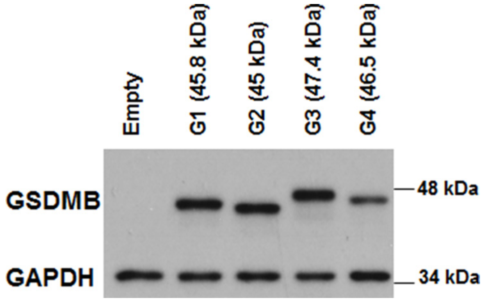

Supplementary Figure S2: Anti-GSDMB antibody detects all GSDMB isoforms. Western blot analysis of GSDMB using our specific monoclonal antibody in HEK293 cells with over-expression of the characterized GSDMB isoforms [35] (G1, NM_001042471.1; G2, NM_018530.2; G3, NM_001165958.1; G4, NM_001165959.1). GAPDH was used as a loading control. Four bands matching the predicted size of each GSDMB isoform were detected. No additional (unspecific) bands were observed.

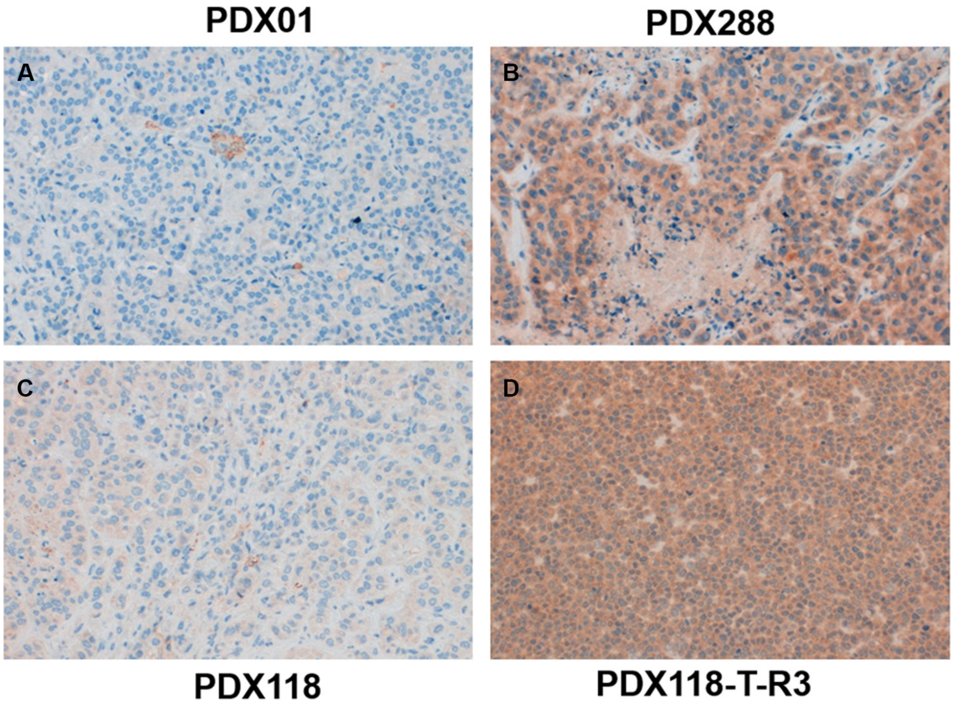

Supplementary Figure S3: Immunohistochemical expression of GSDMB in breast cancer Patient Derived Xenografts (PDX). (**A**) (PDX01): PDX derived from HER2-negative tumor; (**B**) (PDX288) and (**C**) (PDX118): HER2-positive cancers. PDX118 (c) was classified as sensitive and PDX288 (b) as resistant to *in vivo* trastuzumab treatment. (**D**) (PDX118T-R5): trastuzumab-resistant PDX originated from PDX118 (c) by chronic trastuzumab treatment *in vivo*. All the panels are shown at the same magnification x 20.

Supplementary Table S1: High levels of GSDMB mRNA are associated with the HER2-positive phenotype in breast cancer microarray datasets

Dataset	Ur-Rehman 2013 (n = 1570)[a]		TCGA 2012 (n = 526)[b]	
**Parameter**	**GSDMB High**	**GSDMB Low**	**GSDMB High**	**GSDMB Low**
ER +	163/702 (23%)	539/702 (77%)	94/401 (23%)	307/401 (77%)
ER −	81/207 (39%)	126/207 (61%)	38/125 (30%)	87/125 (70%)
X^2 p value	**p = 0.001**		P = 0.125	
PR +	52/231 (22.5%)	179/231 (77.5%)	69/340 (20%)	271/340 (80%)
PR −	12/53 (22.6%)	41/53 (77.4%)	63/186 (34%)	123/186 (66%)
X^2 p value	p = 0.9		**p = 0.001**	
Grade I	17/153 (11%)	136/153 (89%)		
Grade II	84/445 (19%)	361/445 (81%)	N/A	N/A
Grade III	126/372 (34%)	246/372 (66%)		
X^2 p value	**p = 0.001**			
LN +	28/143 (19.6%)	115/143 (80.4%)	77/258 (30%)	181/258 (70%)
LN −	242/946 (26%)	704/946 (74%)	55/268 (20%)	213/268 (80%)
X^2 p value	p = 0.145		**p = 0.016**	
HER2+ IHC	33/63 (52.4%)	30/63 (47.6%)	50/75 (67%)	25/75 (33%)
HER2 − IHC	44/243 (18%)	199/243 (82%)	82/451 (18%)	369/451 (82%)
X^2 p value	**p < 0.001**		**p < 0.001**	
Mol. Subtype (SAM)[c]	HER2 **p < 0.001**		HER2 **p < 0.001**	
ERBB2 Amp[d]			50/67 (75%)	17/67 (25%)
ERBB2 Norm	N/A	N/A	82/459 (18%)	377/459 (82%)
X^2 p value			**p < 0.001**	
GSDMB Amp[d]			48/58 (83%)	10/58 (17%)
GSDMB Norm	N/A	N/A	84/468 (18%)	384/468 (82%)
X^2 p value			**p < 0.001**	

In silico analysis of GSDMB expression was performed in two independent gene expression datasets: [a] The Ur-Rehman dataset [36], a compilation of eight different studies performed on the HG-U133A Affymetrix platform, [b] TCGA (The Cancer Genome Atlas) study [37]. Normalized GSDMB expression was categorized as "high" when it was above the third quartile (top 25% expression) of all tumor samples; otherwise, it was considered "low". Association of GSDMB levels with clinical and pathological features was tested by Chi-square (X^2). [c] Tumors were classified within the different molecular subtypes using the PAM50 classifier [38], and the association of GSDMB-high tumors with any of these types was tested by SAM [39] using ROCK statistical tools.[d] Gene amplification (Amp) assessed by copy number aberration using Affymetrix 6.0 SNP arrays. Norm, normal (not amplified). In all statistical analyses a p value < 0.05 (considered significant) is highlighted in bold letters. ER, estrogen receptor; PR, progesterone Receptor; IHC, immunohistochemistry; N/A, data not available.

Supplementary Table S2: Summary of clinical, pathological and immunohistochemical features of breast carcinomas included in discovery and validation series

	Discovery series	Validation series
	†*n* (%)	†*n* (%)
**Grade**		
1	5/53 (9.4)	11/95 (11.6)
2	25/53 (47.2)	26/95 (27.4)
3	23/53 (43.4)	58/95 (61.1)
**Estrogen Receptor expression**		
Negative	20/53 (37.7)	35/95 (36.8)
Positive	33/53 (62.3)	60/95 (63.2)
**Progesterone Receptor expression**		
Negative	29/52 (55.8)	49/93 (52.7)
Positive	23/52 (44.2)	44/93 (47.3)
**HER2 amplification**		
Negative	24/53 (45.3)	0
Positive	29/53 (54.7)	95 (100)
**pCR***		
Responders	17/29 (58.6)	58/95 (61.1)
Non-responders	12/29 (41.4)	37/95 (38.9)
**Relapse***		
No	21/26 (80.8)	47/66 (71.2)
Yes	5/26 (19.2)	19/66 (28.8)

The data make reference to the available cases for each marker. †*n* (%), number of analyzed cases and (percentage). *pCR: pathological complete response when there is no invasive presence of tumour at the breast or ganglia level and Relapse as local or distant recurrence in HER2-positive tumours.

Supplementary Table S3: GSDMB immunohistochemical expression and gene amplification (FISH) in HER2-positive breast carcinomas (*n* = 95) included in the validation series

	Validation series
	**n* (%)
***GSDMB* amplification (*n* = 95)**	
Negative	37 (38.9)
Positive	58 (61.1)
**GSDMB expression (*n* = 93)**	
Negative	29 (31.2)
Positive	64 (68.8)

**n* (%), number of analyzed cases and (percentage).

Supplementary Table S4: Summary of clinical, pathological and immunohistochemical features of breast carcinomas series treated with adjuvant regimens (*n* = 138)

	†*n* (%)
**Grade (*n* = 138)**	
1	19 (13.8)
2	59 (42.8)
3	60 (43.4)
**Estrogen receptor expression (*n* = 104)**	
Negative	45 (43.3)
Positive	59 (56.7)
**Progesterone receptor expression (*n* = 127)**	
Negative	41 (32.3)
Positive	86 (67.7)
**HER2 amplification (*n* = 129)**	
Negative	76 (58.9)
Positive	53 (41.1)
**GSDMB expression (*n* = 133)**	
Negative	100 (75.2)
Positive	33 (24.8)
**Lymph node metastasis (*n* = 46)***	
Negative	22(44.9)
Positive	27 (55.1)
**Distant metastasis (*n* = 46)***	
Negative	30 (65.2)
Positive	16 (34.8)

*Only evaluated in HER2-positive breast carcinomas. †*n* (%), number of analyzed cases and (percentage).
